# Comprehensive Analysis of the Complete Mitochondrial Genome of *Rehmannia chingii*: An Autotrophic Species in the Orobanchaceae Family

**DOI:** 10.3390/genes15010098

**Published:** 2024-01-15

**Authors:** Ying Han, Yan-Lei Feng, Jie Wang, Shan-Shan Zhu, Xin-Jie Jin, Zhi-Qiang Wu, Yong-Hua Zhang

**Affiliations:** 1 College of Life and Environmental Science, Wenzhou University, Wenzhou 325035, China; hanying_hn@163.com (Y.H.); xinjie_jin@yeah.net (X.-J.J.); 2 College of Agriculture and Biotechnology & ZJU-Hangzhou Global Scientific and Technological Innovation Center, Zhejiang University, Hangzhou 310058, China; fengyanlei@outlook.com; 3 Shenzhen Branch, Guangdong Laboratory of Lingnan Modern Agriculture, Key Laboratory of Synthetic Biology, Ministry of Agriculture and Rural Affairs, Agricultural Genomics Institute at Shenzhen, Chinese Academy of Agricultural Sciences, Shenzhen 518120, China; wangjie@stu.zafu.edu.cn; 4 School of Marine Sciences, Ningbo University, Ningbo 315211, China; zhushanshan1@nbu.edu.cn; 5 State Key Laboratory for Managing Biotic and Chemical Threats to the Quality and Safety of Agro-Products, Ningbo University, Ningbo 315211, China; 6 Institute for Eco-Environmental Research of Sanyang Wetland, Wenzhou University, Wenzhou 325035, China

**Keywords:** mitogenome, *Rehmannia chingii*, Orobanchaceae, organelle genome, RNA editing

## Abstract

*Rehmannia chingii* is an important medicinal plant with immense value in scientific research. However, its mitochondrial genome (mitogenome) has not yet been characterized. Herein, based on whole-genome Illumina short reads and PacBio HiFi reads, we obtained the complete mitogenome of *R. chingii* through a de novo assembly strategy. We carried out comparative genomic analyses and found that, in comparison with the plastid genome (plastome) showing a high degree of structural conservation, the *R. chingii* mitogenome structure is relatively complex, showing an intricate ring structure with 16 connections, owing to five repetitive sequences. The *R. chingii* mitogenome was 783,161 bp with a GC content of 44.8% and contained 77 genes, comprising 47 protein-coding genes (CDS), 27 tRNA genes, and 3 rRNA genes. We counted 579 RNA editing events in 47 CDS and 12,828 codons in all CDSs of the *R. chingii* mitogenome. Furthermore, 24 unique sequence transfer fragments were found between the mitogenome and plastome, comprising 8 mitogenome CDS genes and 16 plastome CDS genes, corresponding to 2.39% of the *R. chingii* mitogenome. Mitogenomes had shorter but more collinear regions, evidenced by a comparison of the organelles of non-parasitic *R. chingii*, hemiparasitic *Pedicularis chinensis*, and holoparasitic *Aeginetia indica* in the Orobanchaceae family. Moreover, from non-parasitic to holoparasitic species, the genome size in the mitogenomes of Orobanchaceae species did not decrease gradually. Instead, the smallest mitogenome was found in the hemiparasitic species *P. chinensis,* with a size of 225,612 bp. The findings fill the gap in the mitogenome research of the medicinal plant *R. chingii*, promote the progress of the organelle genome research of the Orobanchaceae family, and provide clues for molecular breeding.

## 1. Introduction

Plant organelle genomes are believed to have originated from ancient endophytic bacteria [1]. They are semi-autonomous organelles with a transcription/translation system that is not exactly the same as the nuclear genome. The plastid genome (plastome, cp DNA) has a conserved gene number and structure in most plants, making them a good source for phylogenetic studies [2,3,4]. The mitochondrion is an important organelle of eukaryotes, as it is the energy factory of organisms and plays a crucial role in biological physiological activities by participating in energy metabolism, signal transduction, and apoptosis [5,6]. Cytoplasmic male sterility (CMS) is caused by specific mitochondrial mutations in the cytoplasm of plants, and this can be utilized to achieve the hybrid breeding of crops [7].

Unlike the plastome, the mitochondrial genome (mitogenome, mt DNA) exists in almost all eukaryotes, and its size, structure, and gene content vary greatly among species. The configuration of mitogenomes is highly diverse, including linear, single circular, multiple circular, and the coexistence of circular and linear forms in plants [8,9]. Even within the same genus, several configurations exist; for example, there are complex ring and double-ring structures in three species in *Broussonetia* [10]. Mitogenomes in green plants vary in length from tens of kb to 11.7 Mb (in *Larix sibirica*) [11,12,13,14,15]. The main causes of these differences include structural rearrangement mediated by repeat sequences, gene loss or multiple copies of genes, and DNA transfer from internal and external sources [1,14]. The protein-coding sequences (CDSs) of plant mitogenomes are generally highly conserved, usually exhibiting very low nucleotide replacement rates, and the CDS content is independent of the genome size [16]. The “evolutionary paradox” indicates that the non-coding regions of the mitogenome are highly variable. The rearrangement rate of the mitochondrial genomes of plants is generally higher than that of plastomes and animal mitogenomes [17]. The complexity of plant mitogenome structures has resulted in a much narrower research scope compared to that using plastomes [18]. Intramolecular or intermolecular homologous recombination is an important strategy for mitogenome evolution and may be involved in the formation of complex branched structures in mitochondria. Additionally, horizontal gene transfer (HGT) between organelles or cells has contributed to the complexity of plant mitogenomes and serves as a driving force underlying their dynamic evolution [19,20]. Advancements in sequencing technologies (i.e., combining next-generation sequencing and third-generation sequencing technologies) and assembly techniques have provided a strong tool to obtain complete plant mitogenomes, which have markedly accelerated the process of revealing the complexity of plant mitogenomes [21,22]. It is well known that deciphering complete plant mitogenomes is of great significance for understanding the genetic variation, evolutionary mechanism, and breeding of species [7,10,20]

Orobanchaceae Vent., also called the broomrape family, is a large family belonging to the order Lamiales of the eudicots and includes over 2100 species from 102 genera [23,24]. This family has become a model for exploring the evolutionary process of non-photosynthetic plant genomes because it contains autotrophic, hemiparasitic, and holoparasitic forms [25,26]. The basal genera containing *Triaenophora*, *Rehmannia*, and *Lindenbergia* are non-parasitic, and the rest can be grouped as holoparasites or hemiparasites [24]. For plants with different lifestyles, photosynthesis is not of equal importance for obtaining energy; hemiparasitic and holoparasitic plants retrieve nutrients partially or exclusively from their hosts. Therefore, genes that regulate photosynthesis are under different natural selection pressures. The plastomes of Orobanchaceae species have undergone various changes in genome size and structure from autotrophs to heterotrophs, such as pseudogenization, gene loss, and genome rearrangements [26,27]. The nuclear genomes within Orobanchaceae have also experienced evident and convergent gene loss [28]. However, the evolutionary pattern of their mitogenomes remains unknown.

*R. chingii* is a perennial medicinal herb belonging to the genus *Rehmannia* in the family Orobanchaceae [23]. *Rehmannia* contains six species, and *R. chingii* is considered a basal taxon in this genus [28,29]. This species is mainly distributed within Zhejiang province, with partial distribution in the southern part of Anhui and the northern regions of Jiangxi. It is an endemic species of East China [30]. Its medicinal part is the rhizome, which contains abundant bioactive compounds, such as iridoid glycosides [31]. It is known for its effects of clearing heat and cooling blood, nourishing Yin and promoting fluid production, and tonifying the liver and kidneys [32]. In studies of *R. chingii*, the focuses were on plastid genome analyses [28,33], exploring the phylogeography of *R. chingii* using Internal Transcribed Spacer (ITS) sequences and Simple Sequence Repeat (SSR) molecular markers [30], as well as identifying the medicinal components of *R. chingii* [34,35]. However, there are no studies on the mitogenome evolution of *R. chingii*.

In this study, we used a combination of Illumina and PacBio sequencing to assemble the mitogenome of *R. chingii*. We characterized the genomic features of the *R. chingii* mitogenome, including the gene content, RNA editing sites, and codon usage. We also analyzed HGT between the mitogenome and plastome of *R. chingii*, as well as its phylogenetic relationship with organelle systems. Furthermore, we compared the mitogenomes with those of other Lamiales plants, especially those belonging to the Orobanchaceae family. This study provides valuable genetic resources for the evolutionary and functional research of the medicinal plant *R. chingii* whilst advancing the progress in research on *Rehmannia* organelle genomes and providing clues for mitochondrial genomic studies on plants of the Orobanchaceae family.

## 2. Materials and Methods

### 2.1. Plant Materials and Sequencing

One *R. chingii* sample examined in this study was collected from Tianmu mountain, Zhejiang Province, China (30°19′34.06″ N, 119°26′32.88″ E). Fresh leaves and tissue samples were collected, washed with ultra-pure water, immediately frozen with liquid nitrogen, and stored in a −80 °C ultra-low-temperature refrigerator (eppendorf CryoCube F570-86, Molden, Essex, UK). Total DNA was extracted from the young leaves of *R. chingii* by using a modified CTAB method [36], and RNAs were extracted from five tissue samples (roots, stem, leaf, shoot tip, and flower) using an RNAprep Pure Plant Kit (Polysaccharides & Polyphenolics-rich) (Beijing, China); then, we used a NanoDrop One Microvolume UV-Vis Spectrophotometer (Thermo Fisher Scientific, Waltham, MA, USA) to measure the quality of DNA and RNA. Paired-end sequencing was performed on a high-throughput sequencing platform, Illumina HiSeq6000, with a read length of 150bp. Long-read sequencing was conducted using the third-generation sequencing technology SMRT on the PacBio platform. Tissue-specific RNA sequencing libraries were generated using an NEBNext^®^ Ultra™ RNA Library Prep Kit for Illumina^®^ (#E7530L, NEB, Ipswich, MA, USA) following the manufacturer’s recommendations and were sequenced on the Illumina platform. All of the above sequencing protocols were performed by Wuhan Benagen Technology Company.

### 2.2. Assembly and Annotation of Organelle Genomes

Mitochondria were relatively abundant within cells. First, random sampling was performed using SeqKit v2.2.0 [37] on Illumina whole-genome paired-end sequencing data, resulting in the generation of a 10 Gb mitochondrial assembly dataset. To obtain an accurate mitogenome, the extracted paired-end sequencing dataset was assembled using SPAdes v3.13.1 [38] with a de novo assembly strategy. The K-values were set to 67, 87, 97, and 107, resulting in the assembly of scaffolds representing the mitogenome. We downloaded the protein-coding gene sequences of the mitogenome of a Lamiaceae species, *Salvia miltiorrhiza* (NC_023209.1), from the NCBI database (National Center for Biotechnology Information) as a reference. We performed BLAST v2.9.0-2 [39] alignment between these CDS sequences and the assembled scaffolds. Based on the results of BLAST v2.9.0-2 and the assembly depth criteria (nuclear genome coverage < 10×, plastome coverage > 100×), we used Bandage v0.8.1 [40] to filter out mitogenome segments and obtained the mitogenome sequence assembled from the Illumina sequencing data. The mitochondrial sequence was used to perform homology sequence retrieval on the whole-genome HiFi reads (43 Gb) of *R. chingii* using Minimap2 v2.25 [41]. The collected HiFi reads were used for de novo assembly using Flye v2.9.2-b1786 [42], and two rounds of correction were performed using Plion v1.24 [43] based on paired-end sequencing data. We finally obtained the *R. chingii* mitogenome that was adjusted using Bandage v0.8.1. The above process of assembling the complete mitogenome is similar to that of the software GSAT v1.11 [21], but our assembly results had a higher data coverage and were more streamlined (with fewer plastid fragments). Based on the dual-end sequencing data from the second-generation Illumina platform, we used GetOrganelle v1.7.5.3 [44] to assemble the *R. chingii* chloroplast genome. The K-mer values were set to the five levels of 21, 45, 65, 85, and 105. After the assembly was completed, we obtained a complete quadripartite structure of the plastome at the contig level. 

Long-read datasets help obtain the mitogenome. However, the assembly results may only represent the dominant configuration of the *R. chingii* mitochondria due to the structural variability of the mitogenome. These scaffolds may be involved in mediating genome recombination, leading to cryptic configurations. To validate the accuracy of the finally obtained single circular structure, we mapped the long-read sequencing data onto these scaffolds. We expanded an additional 1Kb region beyond the fan-shaped region of each scaffold that mediates recombination to ensure that the mapped long reads fully spanned the repetitive regions.

Using the plant plastome database, CPGAVAS2 [45] was used to annotate the *R. chingii* plastome. We used the CDSs of closely related species, namely, *Rehmannia glutinosa* (OM397952.1), *Castilleja patrramensis* (NC_031806.1), and *S. miltiorrhiza* (NC_023209.1), as references to annotate the *R. chingii* mitogenome using GeSeq [46]. tRNAscan-SE [47] was utilized for tRNA gene annotation, and we manually adjusted and corrected the annotation results of the organelle genome. We generated a genomic map using OGDRAW [48].

### 2.3. Repeat Sequence Detection and Codon Analysis

The REPuter [49] online program was used to detect discrete repeat sequences (DRSs) with the following settings: Minimum Repeat Size = 30 bp, Maximum Computed Repeats = 5000, Hamming distance = 3 (sequence consistency ≥ 90%), and e-value cut-off = 1 × 10^−5^. Tandem Repeats Finder [50] was used to identify tandem repeat sequences (TRSs) with default parameter settings. The detection of Simple Sequence Repeats (SSRs) was performed using misa.pl [51], and the minimum number of repeat units for mononucleotide, dinucleotide, trinucleotide, tetranucleotide, pentanucleotide, and hexanucleotide SSRs were set to 10, 5, 5, 3, 3, and 3.

We extracted the CDS from the mitogenome using Geneious Prime 2021. CodonW v1.4.2 [52] was used to analyze the codon usage of CDSs and calculate the Relative Synonymous Codon Usage (RSCU) values. Codons with RSCU values greater than 1 were defined as optimal codons.

### 2.4. RNA Editing Site Analysis

Based on the transcriptome sequencing data of the root, stem, leaf, shoot tip, and flower tissues of *R. chingii*, we analyzed and statistically evaluated the RNA editing sites in the *R. chingii* organellar genome. The sequencing raw data were filtered using Fastp v0.12.4 [53] during postprocessing. High-quality clean data were mapped to our assembled high-quality mitochondrial and plastome. We used SAMtools v1.3.1 [54] to obtain SNPs and RNA editing sites. After excluding false-positive sites (--max-missing 0.5), we conducted an analysis on RNA editing sites [55].

### 2.5. Transfer Fragments and Collinearity Analysis

To identify transfer fragments between the mitochondrial and plastomes, BLAST v2.9.0-2 was used to search homologous blocks between the organelle genomes of *R. chingii* (with a minimum identity of 80%, an e-value cutoff of 1 × 10^−5^, and fragments longer than 100 bp). We set the repetitive fragments as unique to ensure accurate detection. Simultaneously, we retrieved intergenic homologous regions of the organelle genomes for the non-parasitic species *R. chingii*, the hemiparasitic species *P. chinensis*, and the holoparasitic species *A. indica*. This was carried out to reveal the organelle genome homology among the different types of parasitic plants. TBtools [56] was used to visualize the results.

### 2.6. Organellar Phylogenetical Inference

We downloaded the mitochondrial and plastid genome sequences of the following Lamiales species from the NCBI database: *A. indica* (NC_069194.1 [57], MN529629.1 [58]), *P. chinensis* (NC_072955.1, OQ842968.1), *Pedicularis kansuensis* (NC_072932.1, OQ587613.1), *Castilleja patrramensis* (NC_031806.1, NC_031805.1), *R. glutinosa* (OM397952.1, NC_034308.1 [59]), *S. miltiorrhiza* (NC_023209.1, NC_020431.1 [60]), *Utricularia reniformis* (NC_034982.1, NC_029719.2), *Dorcoceras hygrometricum* (NC_016741.1, NC_016468.1) [61], *Aragoa cleefii* (OK514182.1 [62], MW877562.1 [63]), and *Osmanthus fragrans* (NC_060346.1, NC_042377.1 [64]). We also manually annotated and corrected any annotation errors in these sequences. All genes from each plastid genome and the shared mitochondrial CDSs of *R. chingii* and 10 downloaded species were aligned and concatenated, respectively, in Geneious Prime 2021 after passing incongruence length difference (ILD) in PAUP v4.0 [65] and the substitution saturation test in DAMBE v5.2 [66]. Maximum likelihood (ML) analyses were run in IQ-Tree2 [67] with 1000 bootstrap (BS) replicates. The best-fit nucleotide substitution model (GTR+R6) was identified through ModelFinder v1.6.8 [68]. The final phylogenetic trees were visualized in Figtree v1.4 (http://tree.bio.ed.ac.uk/software/figtree/, accessed on 18 June 2023).

## 3. Results

### 3.1. Genome Assembly and Characterization

**Figure 1:**
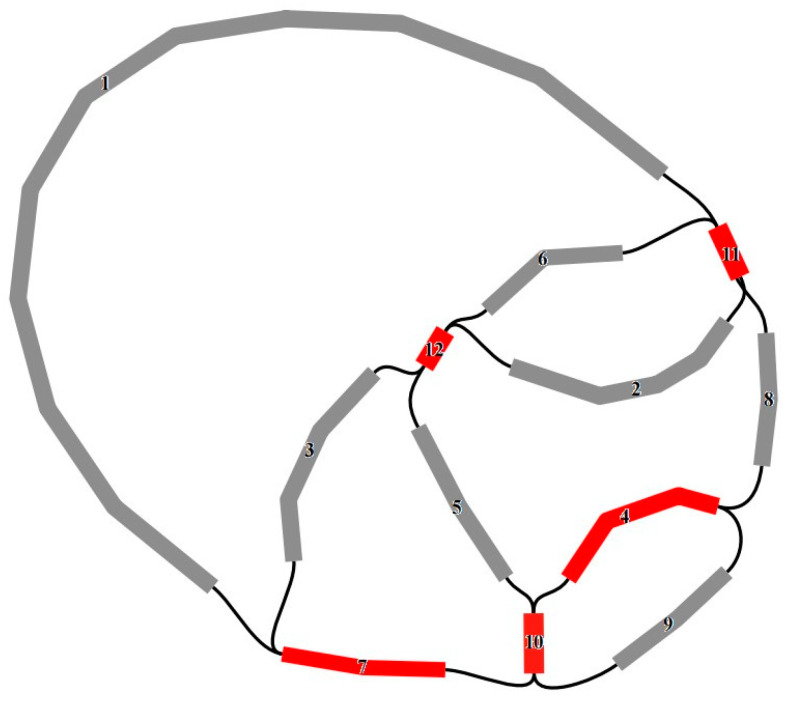
Mitogenome assembly graph and possible connections (black lines) mediated by repeats for *R. chingii*. Unique and repeated contigs are separately colored in gray and red.

We combined the sequencing data from the Illumina and PacBio platforms and successfully assembled an accurate mitogenome of *R. chingii* (Figure 1). We used SPAdes v3.13.1 to perform de novo assembly and obtained 686,450 scaffolds with a cumulative length of 270,674,265 bp. Eighty-two scaffolds were selected as the mitochondrial assembly dataset for the assembly of the third-generation HiFi sequencing data. A higher-quality and higher-depth mitogenome assembly was obtained, comprising 12 scaffolds with a total length of 652,331 bp. The assembled mitogenome displayed a closed, network, and complex molecular structure, with an average depth of 356.3× (Figure 1 and Table S1). The *R. chingii* mitogenome produced 16 connections mediated by five repetitive sequences, all of which were validated by read alignment (Table S2). Each connection method received support. Finally, we disassembled the obtained complex circular structure mediated by repetitive sequences into a single, circular mitogenome. However, there was no unique way to disassemble it.

**Figure 2:**
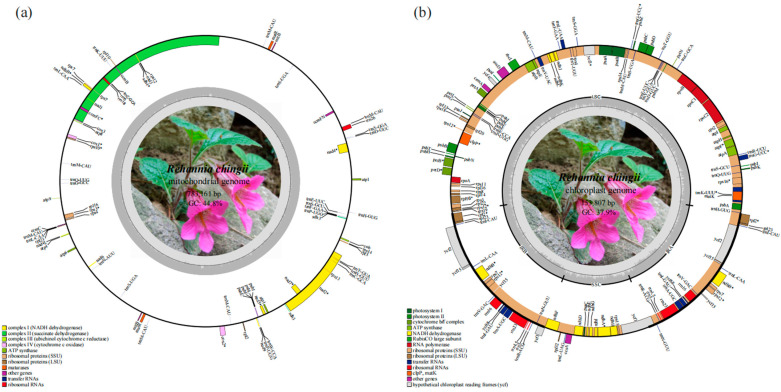
Organelle genome diagrams of *R. chingii*. (**a**) Mitogenome diagram of *R. chingii*. (**b**) Plastome diagram of *R. chingii*.

The *R. chingii* mitogenome had a length of 783,161 bp and a GC content of 44.8%. The base composition was as follows: A: 27.6%, T: 27.6%, G: 22.5%, and C: 22.3%. The *R. chingii* mitogenome contained 77 genes, comprising 47 protein-coding genes, 27 tRNA genes, and 3 rRNA genes (Table 1). The protein coding region of the *R. chingii* mitogenome was 38,484 bp in length with 42.8% GC content. These genes could be further categorized into 12 specific functional classes (Table 1 and Figure 2a). Among the 38 unique mitogenome CDSs, four genes (*atp*1, *sdh*3, *mtt*B, and *mat*R) had two copies, and they also contained five plastid transfer CDSs (*ndh*B, *psb*E, *psb*F, *psb*J, and *psb*L). The genes *rps*3, *rps*10, *cox*1, *cox*2, and *ccm*Fc contained one intron each. The genes *nad*4 and *nad*7 contained three introns, while *nad*1, *nad*2, and *nad*5 contained four introns each. Five tRNA genes (*trn*C-GCA, *trn*L-CAA, *trn*P-UGG, *trn*M-CAU, and *trn*S-UGA) exhibited the multicopy phenomenon (Table 1). The prediction of the tRNA protein secondary structure showed that most tRNAs could be folded into a typical cloverleaf form, except for *trn*F-GAA, *trn*L-CAA, and *trn*N-AUU. The assembled plastome of *R. chingii* had a total length of 153,807 bp and contained 134 genes (Figure 2b), comprising 89 protein-coding genes, 37 tRNA genes, and 8 rRNA genes; the GC content was 37.9%.

### 3.2. Codon and Repeat Sequence Analysis

**Figure 3:**
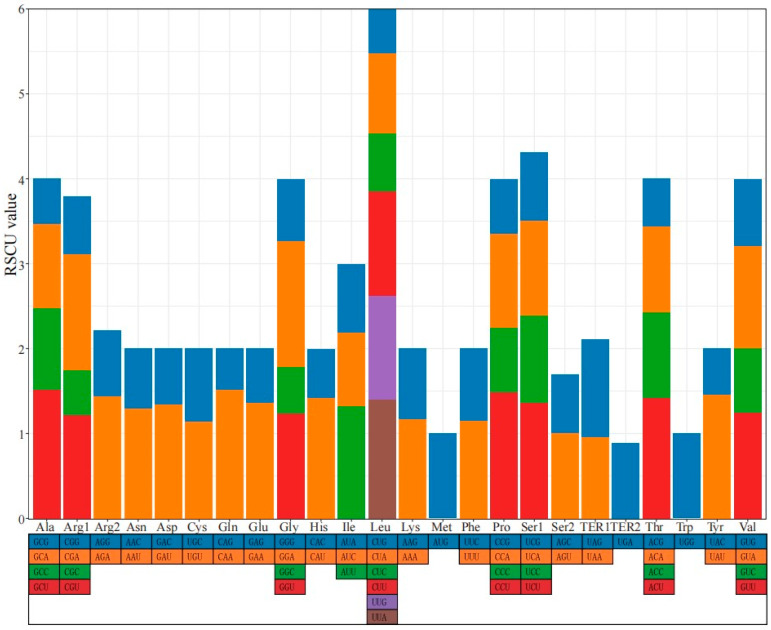
Codon usage of the *R. chingii* mitochondrial CDSs.

In the *R. chingii* mitogenome, most CDSs began with the codon ATG and ended with TAA or TGA. There were 12,828 codons in the CDSs of the *R. chingii* mitogenome. The most and least frequently used amino acids were leucine (Leu) and cystine (Cys), accounting for approximately 10.58% and 1.40%, respectively. The codon UUU, which encodes phenylalanine (Phe), was the most frequently used codon, with a frequency of 3.74%. Its RSCU value was 1.15. The codon CGC, which encodes arginine (Arg), had the lowest frequency of 0.58%. Its RSCU value was 0.53. The RSCU analysis revealed that 30 codons had values greater than 1, accounting for 46.88% of the total codons (Figure 3 and Table S3).

By using online program, namely, REPuter and Tandem Repeats Finder, 163 forward repeat sequences, 125 palindromic repeat sequences, and 11 TRSs were detected in the *R. chingii* mitogenome (Figure S1a). Furthermore, 67 SSR loci were detected, with 53 loci belonging to the mononucleotide repeat type and only 1 locus belonging to the trinucleotide repeat type (Figure S1b). No tetranucleotide, pentanucleotide, or hexanucleotide repeat types were detected. Among the mononucleotide repeat types, A/T repeats were the most common. Among the dinucleotide repeat types, AA/TT repeats were the most abundant.

### 3.3. RNA Editing Site Analysis

**Figure 4:**
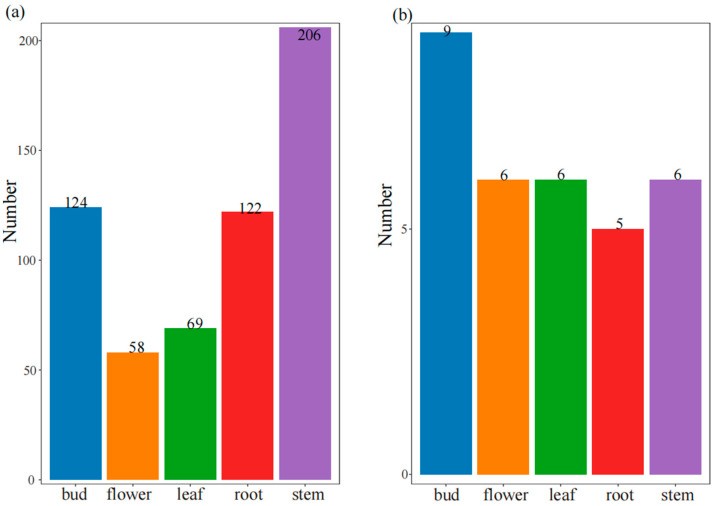
RNA editing sites of the *R. chingii*. (**a**) RNA editing sites in CDS region of mitogenome. (**b**) RNA editing sites in the CDS region of plastome.

RNA editing events are widely present in higher plants and are necessary for the expression of their mitochondrial genes [55]. We found 579 RNA editing sites in mitochondrial CDS and 32 sites in plastid CDS. The number of RNA editing sites in the *R. chingii* mitogenome was approximately higher than that in its plastid genome (Figure 4). In contrast to the plastid genomes, RNA editing events were prevalent in the mitochondrial genomes. The C>T type dominated RNA editing sites, and more RNA editing sites were found in root, stem, and shoot tissues. Among them, mitogenome RNA editing sites were generally abundant across five tissues, with more than 50 sites in each tissue, while plastome RNA editing sites were mostly detected in bud tissues with 9 sites.

### 3.4. Transfer Fragments Detection and Collinearity Analysis

**Figure 5:**
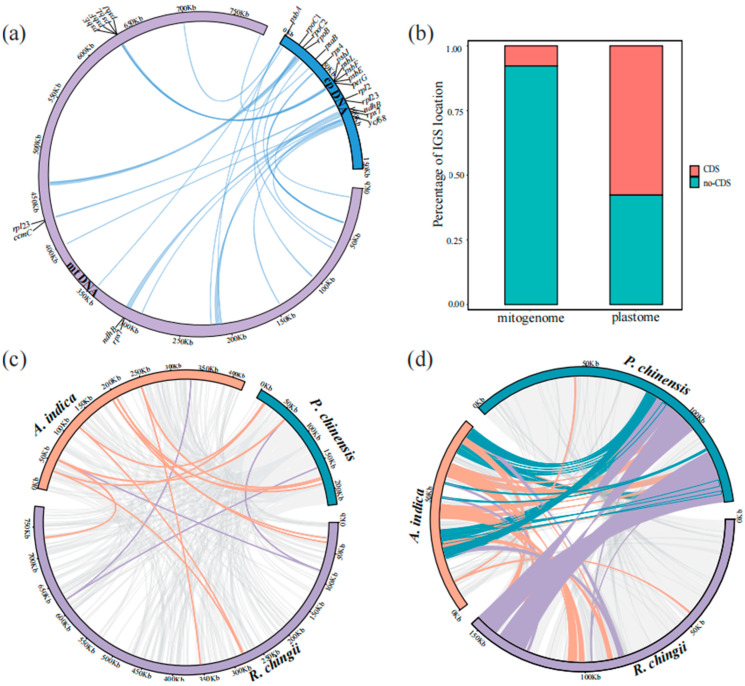
Synteny of organelle genomes. (**a**) MTPTs between organelle genomes of *R. chingii*. (**b**) MTPT ratio in coding region and non-coding region. (**c**) Homologous segments of mitogenomes in *R. chingii*, *A. indica,* and *P. chinensis*. (**d**) Homologous segments of plastomes in *R. chingii*, *A. indica,* and *P. chinensis*.

We used the BLAST v2.9.0-2 program to identify sequence transfers between organelles, and 24 unique transfer fragments were obtained between the mitogenome and plastome of *R. chingii*. These transfer fragments accounted for 2.39% of the mitogenome and 12.16% of the plastome. Among these transfer fragments, 8 mitogenome CDS genes and 16 plastome CDS genes were identified (Figure 5a and Table S4). Furthermore, the transfer frequency of the coding sequences in the plastome was higher than that in non-coding regions, while the integration of non-coding regions in the mitogenome was dominant (Figure 5b). *rps*7, *rpl*23, *ndh*B, *psb*E, *psb*F, *psb*J, and *psb*L CDSs were simultaneously annotated in the transfer fragments.

Furthermore, a comparative analysis of the two organelle genomes of non-parasitic *R. chingii*, hemiparasitic *P. chinensis*, and holoparasitic *A. indica* revealed significant collinearity (Figure 5c,d). The homologous segments of *P. chinensis* and *R. chingii* accounted for more than 90% of the total plastome length, and those of *A. indica* accounted for 65.05% of the total length. The homologous segments of the mitogenome of *A. indica* and *R. chingii* accounted for 26.05% and 22.39% of the total length respectively, while those of *P. chinensis* accounted for 64.15% of the total length. Comparing three species with different modes of tropism, mitogenomes were found to have shorter but more collinear regions. A total of 358 homologous segments were identified among their mitogenomes, with 33 shared genes (Figure 5c; Table S5). In contrast, plastomes had fewer collinear regions but with longer lengths, with a total of 142 homologous segments with 21 shared genes (Figure 5d; Table S5).

### 3.5. Phylogenetic Analysis of R. chingii

**Figure 6:**
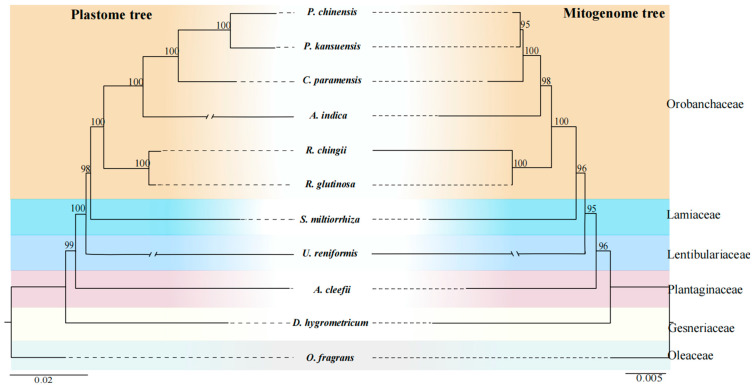
Phylogenetic analysis. ML tree based on plastid genes, except for duplicates (left) and 27 shared mitochondrial genes (right). Numbers near nodes indicate the bootstrap value. Colors show the families.

We performed a phylogenetic analysis using the shared CDSs of the mitogenomes, all genes from the plastomes of *R. chingii,* and 10 other Lamiales species. Twenty-seven shared CDSs were obtained from these mitochondrial genomes, namely, *atp*1, *atp*4, *atp*6, *atp*8, *atp*9, *ccm*B, *ccm*C, *ccm*Fc, *ccm*Fn, *co*b, *cox*1, *cox*2, *cox*3, *mat*R, *nad*1, *nad*2, *nad*3, *nad*4, *nad*4L, *nad*5, *nad*6, *nad*7, *nad*9, *rps*3, *rps*4, *rps*12, and *rps*13. The aligned matrix in the mitogenome was 29,713 bp, within 3023 variable sites, comprising 2363 singleton variable sites and 660 parsimony informative sites. In the plastome, the aligned matrix was 82,350 bp, within 15,064 variable sites, comprising 10,599 singleton variable sites and 4465 parsimony informative sites (Table S6). The phylogenetic relationships revealed by the mitochondrial CDSs were consistent with those of the plastid genes. In the mitogenome tree, all nodes had supported values (BS) greater than 95, while in the plastome tree, most nodes were strongly supported by BS = 100, with only two nodes with BS = 99 and 98 (Figure 6). All species belonging to the Orobanchaceae family were grouped; that is, two *Rehmannia* species (*R. chingii* and *R. glutinosa*) were clustered into the basal subclade with a bootstrap value of 100% and formed a sister subclade to the subclade of four other Orobanchaceae species (*A. indica*, *C. paramensis*, *P. kansuensis*, and *P. chinensis*) (Figure 6). Both the mitogenome and plastome trees of all 11 Lamiales species showed *O. fragrans* (Oleaceae) as the basal taxon, followed by *D. hygrometricum* (Gesneriaceae), *A. cleefii* (Plantaginaceae), *U. reniformis* (Lentibulariaceae), *S. miltiorrhiza* (Lamiaceae), and six Orobanchaceae species.

### 3.6. Comparison of Genomic Features with Ten Other Lamiales mitogenomes

**Figure 7:**
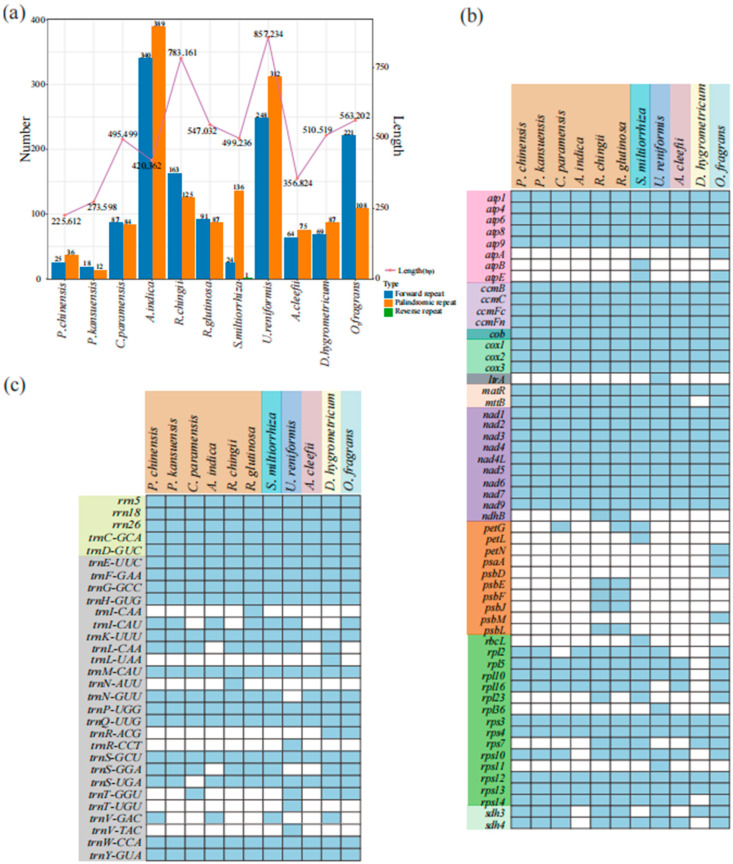
Comparison of genome size, dispersed repeats, and gene content in 11 Lamiales mitogenomes. (**a**) Genome size and dispersed repeats. The line shows the mitogenome size of the related species (**b**) CDS gene. (**c**) RNA gene. The blue boxes represent active genes, and the blank boxes represent missing genes.

We compared the genome size, dispersed repeats, and GC content of *R. chingii* with 10 other Lamiales mitogenomes (containing 6 Orobanchaceae species). The size of these 11 mitogenomes varied greatly, ranging from 225,612 bp (*P. chinensis*) to 857,234 bp (*U. reniformis*) (Figure 7a; Table S7). Of these Orobanchaceae species, the biggest genome size was from the non-parasitic genus *Rehmannia* (*R. chingii*: 783,161 bp; *R. glutinosa*: 547,032 bp), followed by the hemiparasitic genus *Castilleja* (*C. paramensis*: 495,499 bp) and the holoparasitic *Aeginetia* (*A. indica*: 420,362 bp). The smallest one was from the hemiparasitic genus *Pedicularis* (*P. kansuensis*: 273,598 bp; *P. chinensis*: 225,612 bp) (Figure 7a; Table S7). The dispersed repeats were mainly forward and palindromic repeats in the Orobanchaceae species and the rest of the Lamiales mitogenomes, with only one reverse repeat in *S. miltiorrhiza* (Figure 7a). Except for *A. indica*, the number of dispersed repeats of the remaining species correlated strongly with their genome size (Figure 7a). Only 30 dispersed repeats were identified in the smallest mitogenome (hemiparasitic *P. kansuensis*, 273,598 bp), and 729 were identified in the largest (holoparasitic *A. indica*, 420,362 bp). In contrast, they showed a relatively similar GC content, ranging from 43.3 to 45.0% (Orobanchaceae species: 43.5–45%) (Table S7).

By comparing the mitogenomes of these 11 Lamiales species, we found differences in their gene contents, ranging from 55 to 77 genes (6 Orobanchaceae species: 55–77 genes) (Table S7). Most of the mitochondrial protein-coding genes and rRNA genes were highly conserved, and *mtt*B was absent only in *D. hygrometric*. Transfer genes from plastids, such as *atp*A, *atp*B, *atp*E, *ndh*B, *pet*G, and *pet*L (Figure 7b, orange region), were only identified in *R. glutinosa*, *R. chingii*, *S. miltiorrhiza*, and *O. fragrans*. *rps*7 was completely lost in the holoparasitic and semi-parasitic species of the family Orobanchaceae, and Complex II genes (*sdh*3 and *sdh*4) in holoparasitic *A. indica* were completely lost. *rps*11 and *ltr*A were only found in *U. reniformis.* Twelve tRNA genes were found in these 11 species, and, notably, the loss of tRNA genes occurred more frequently (Figure 7c).

## 4. Discussion

### 4.1. Characterization of the R. chingii Mitogenome

In this study, we assembled a gap-free, circular, complex multi-component map of the complete mitogenome of *R. chingii* by combining data from second- and third-generation sequencing analyses (Figure 1). Via sequence alignment, we confirmed the presence of 16 different connectivity patterns mediated by five repetitive sequences, further demonstrating that the mitogenome may have experienced dynamic evolution. This result also suggests that the *R. chingii* mitogenome may have multiple branching conformations, explaining why our assembly result was not a single circular conformation. Previous studies have indicated that the plant mitogenome is not a simple single-molecule structure but a complex multi-component structure, typically owing to repetitive sequences [1,69,70]. Additionally, these repetitive sequences are the main reason for the large differences in the mitogenome size among different plants and a major contributor to the presence of mitogenome isomers. For example, in studies of mitogenomes in the Rosaceae family, the size of the mitogenome was found to be correlated with the number and length of repetitive sequences [71]. The mitogenome is usually larger than the plastome in plants but contains fewer genes than the plastome, mainly due to several non-coding sequences in the mitogenome [70]. In our study, the mitogenome of *R. chingii* was 783,161 bp long, containing 77 genes, and its plastome was 153,807 bp long, with 134 genes. Among them, the CDS in the mitogenome accounted for 4.91%, while in the plastome, it accounted for 51.73%. The plant mitogenome was conserved in the number, type, and sequence of functional genes, but the position and arrangement of the mitogenome varied in different species [72]. This may be related to large recombination events during the evolution of the repeated sequences present in the *Rehmannia* mitogenome.

### 4.2. MTPTs in the R. chingii Mitogenome

Intracellular horizontal gene transfer (IGT) refers to a type of sequence migration among the mitogenome, plastome, and nuclear genome [73,74]. The most common phenomenon is the integration of DNA fragments from plastids to mitochondrial genomes (MTPTs) [75,76]. Eight complete CDSs (*rps*7, *rpl*23, *ndh*B, *psb*J, *psb*L, *psb*F, *psb*E, and *pet*G) were found to migrate from the plastome to the mitogenome in *R. chingii* and to some other plastid gene fragments. Remarkably, seven genes (*rps*7, *rpl*23, *ndh*B, *psb*E, *psb*F, *psb*J, and *psb*L) were simultaneously annotated in both the mitogenome and plastome; however, other genes transferred from the plastid might have undergone pseudogenization in the mitogenome [77,78]. In angiosperms, it is common for tRNA genes to transfer from the plastome to the mitogenome [79]. This phenomenon has also been observed between the organelle genomes of *R. chingii*, such as *trn*S-GGA and *trn*L-CAA (Table S4). A collinearity analysis showed that the collinearity regions among the plastomes of the three Orobanchaceae species (*R. chingii*, *A. indica*, and *P. chinensis*) accounted for the majority of the sequences, despite the abnormally short plastome in the holoparasitic *A. indica* (Figure 5d). However, the collinear regions among the mitogenomes constituted only a small part of the genomes, although the collinear regions in the mitogenomes were longer than those in the plastomes in these three species. Therefore, the retained plastomes in the Orobanchaceae family might exhibit a relatively conserved structure, while the mitogenomes show significant heterogeneity due to active repeat-mediated recombination and horizontal gene transfer (HGT) events during plant evolution [57,80].

### 4.3. RNA Editing in the R. chingii Mitogenome

RNA editing events are highly frequent in plant mitochondrial genomes and are critical for gene expression [10,81]. RNA editing sites in different plant mitogenomes exhibit certain variations. For instance, the mitogenome of *Arabidopsis thaliana* shows 441 RNA editing sites in 36 CDSs, while the tea plant (*Camellia sinensis* var. *Assamica* cv. *Duntsa*) has 536 RNA editing sites in 47 CDSs [15,82]. In this study, 579 RNA editing sites were detected in 47 CDSs of the *R. chingii* mitogenome. There was great heterogeneity in the abundance and types of RNA editing sites of organelle genome among the different tissues of *R. chingii*. The stem possessed the greatest number of RNA editing sites in the mitogenome, while the root had the greatest number of RNA editing sites in the plastome in *R. chingii*. The C>T type was dominant in both organelle genomes and in five tissues. These results suggest that cellular functional differentiation may be the primary reason for the heterogeneity in RNA editing site distribution among the different tissues. RNA editing events have a potential function in the development of plant cytoplasmic male sterility [83], and RNA editing in functional genes can lead to amino acid changes [84,85]. 

### 4.4. Phylogenetic Relationships and Comparison of Genomic Features in Orobanchaceae Mitogenomes

Unlike plastid and nuclear genomes, mitogenomes are rarely used in phylogenetic analyses of higher plants due to the low mutation rate, frequent genome rearrangement, and foreign DNA integration [16,86,87,88]. In this study, based on the available mitogenome data (Figure 6), we performed phylogenetic analyses within Orobanchaceae, as well as within Lamiales. Notably, these results were almost fully consistent with the plastome results, as well as congruent with the phylogeny of Lamiales species described in the APG IV system [23]. These results suggest that some conserved gene clusters in plant mitogenomes can be used as signals for phylogenetic analyses.

In our study, although the largest mitogenome size was from the non-parasitic species *R. chingii* (783,161 bp), the smallest one was from the hemiparasitic species *P. chinensis* (225,612 bp) instead of the holoparasitic species *A. indica* (420,362 bp). Therefore, from autotrophs (non-parasitic species) to heterotrophs (holoparasitic species), the genome size in the mitogenomes of Orobanchaceae species did not decrease gradually (Figure 7a), unlike the results obtained from the plastomes [26] and nuclear genomes [28]. Three species (*R. chingii*, *P. chinensis*, and *A. indica*) with different trophic modes had shorter collinear regions in their mitogenomes than in their plastomes (Figure 5c,d), indicating that Orobanchaceae species might have a weaker relationship in their collinearity in mitogenomes. The *rps*7 gene is known to encode ribosomal protein S7, and *rps*10 codes for the ribosomal protein S10 (https://www.ncbi.nlm.nih.gov/gene/, accessed on 10 January 2024), both of which are important ribosomal protein genes. For gene loss, compared to the non-parasitic genus *Rehmannia*, *rps*7 was completely lost in holoparasitic *A. indica* and semi-parasitic species (namely, *C. paramensis*, *P. kansuensis*, and *P. chinensis*). Complex II genes (*sdh*3 and *sdh*4) and *rps*10 in holoparasitic *A. indica* were completely lost; these genes are known to be involved in mitochondrial energy activity, which may also be one of the reasons why *A. indica* is a parasitic plant. In addition, *R. chingii* had one more *rpl*23 gene than *R. glutinosa*, which encoded the ribosomal protein L23. The effects of these gene loss phenomena on mitogenome function and evolution need to be further explored. However, due to insufficient sampling, this study could not provide adequate genetic information for understanding the evolutionary clues of Orobanchaceae mitogenomes. Future studies require more extensive sampling.

## 5. Conclusions

In summary, this study employed a combined assembly strategy using long and short-read sequencing data to assemble and annotate the organelle genomes of *R. chingii*, resulting in high-quality organelle genomes of *R. chingii*. The abundance of RNA editing sites in the organelle genomes of *R. chingii* exhibited uneven distribution across different tissues, with the majority of RNA editing events occurring within the CDSs. We also conducted a comprehensive comparison of the organelle genomes of *R. chingii* and identified MTPTs. The findings are expected to provide genetic resources for studying gene transfers between mitochondria and plastids. Moreover, the utilization of long-read sequencing enabled us to better decipher the complex structure of the *R. chingii* mitogenome, particularly the dynamic transformation of plant mitogenomes. Finally, the comprehensive analysis of the Orobanchaceae organelle genomes has advanced our understanding of the Orobanchaceae mitogenome structure and evolution.

## Figures and Tables

**Table 1:** Known functional genes in the *R. chingii* mitogenome.

Group of Genes	Name of Genes
Complex I	*nad*1 *, *nad*2 *, *nad*3, *nad*4 *, *nad*4L, *nad*5 *, *nad*6, *nad*7 *, *nad*9
Complex II	*sdh*3 (×2), *sdh*4
Complex III	*cob*
Complex IV	*cox*1 *, *cox*2 *, *cox*3
Complex V	*atp*1 (×2), *atp*4, *atp*6, *atp*8, *atp*9
Cytochrome c biogenesis	*ccm*B, *ccm*C, *ccm*Fc *, *ccm*Fn
Maturases	*mat*R (×2)
Transport membrane protein	*mtt*B (×2)
Ribosome	*rpl*2, *rpl*5, *rpl*10, *rpl*16, *rpl*23, *rps*3 *, *rps*4, *rps*7, *rps*10 *, *rps*12, *rps*13, *rps*14
rRNA	*rrn*5, *rrn*18, *rrn*26
tRNA	*trn*C-GCA (×2), *trn*D-GUG, *trn*E-UUC, *trn*F-GAA, *trn*G-GCC, *trn*H-GUG, *trn*K-UUU, *trn*L-CAA (×2), *trn*M-CAU (×6), *trn*N-AUU, *trn*N-GUU, *trn*P-UGG (×2), *trn*Q-UUG, *trn*S-GCU, *trn*S-GGA, *trn*S-UGA (×2), *trn*W-CCA, *trn*Y-GUA
plastid gene	*ndh*B *, *psb*E, *psb*F, *psb*J, *psb*L

(×) gene number; * with intron(s).

## Data Availability

The entire complete mitogenome sequence with gene annotation has been submitted to NCBI GenBank under accession number OR601177. The entire complete plastome sequence with gene annotation has been submitted to NCBI GenBank under accession number OR601178. The original sequencing data have been uploaded to the China National GeneBank DataBase (CNGBdb). The project number is CNP0005183, CNX0941038 is the raw data from the Illumina sequencing for mitogenome assembly, and CNX0941039 is the raw data from PacBio sequencing for mitogenome assembly.

## Supplementary Materials

The following supporting information can be downloaded at https://www.mdpi.com/article/10.3390/genes15010098/s1, Figure S1: Repeat analysis of *R. chingii* mitogenome. Table S1: Results of the *R. chingii* mitogenome. Table S2: Verification of 16 connection relationships in the *R. chingii* mitogenome. Table S3: The codon uses and the RSCU value in the *R. chingii* mitogenome. Table S4: Specific characteristics of MTPTs in the *R. chingii* mitogenome. Table S5: Shared genes and endemic genes in the three species (*R. chingii*, *P. chinensis*, *A. indica*). Table S6: Characterization of the sequence matrices used for organellar phylogenetic inference. Table S7: Characterizations of mitogenomes of 11 Lamiales species.
