# Supplementary material for: Comprehensive Analysis of the Complete Mitochondrial Genome of Rehmannia chingii: An Autotrophic Species in the Orobanchaceae Family
Source: Genes (Basel). 2024 Jan 15;15(1):98. doi: 10.3390/genes15010098 (PMC10815111; doi:10.3390/genes15010098)
Supplement: Supplementary file 1 [file genes-15-00098-s001.zip › genes-2807561-supplementary.pdf]

Table S1 Results of the *R. chingii* mitogenome

Scaffold	Length(bp)	Depth(×)	Scaffold	Length(bp)	Depth(×)
1	249,921	360.0	7	33,548	222.0
2	90,804	399.0	8	28,618	327.0
3	59,096	353.0	9	27,917	283.0
4	55,758	372.0	10	14,750	636.0
5	41,969	311.0	11	10,655	653.0
6	38,023	254.0	12	1,272	661.0

Table S2 Verification of 16 connection relationships in the *R. chingii* mitogenome

Lable	Length	Average pident	Toal scaffold (>1000bp)	scaffolds support	E value < 1e-5
4_8	56,758	99.70%	501	10(score)	100%
4_9	56,758	99.70%	501	10(score)	100%
7_1	34,548	99.78%	502	1(sacffolds)	100%
7_3	34,548	99.77%	310	20(score)	100%
10_4	15,750	99.71%	500	65(scaffolds)	100%
10_5	15,750	99.72%	500	77(sacffolds)	100%
10_7	15,750	99.72%	500	88(scaffolds)	100%
10_9	15,750	99.72%	500	71(scaffolds)	100%
11_1	11,655	99.77%	500	217(scaffolds)	100%
11_2	11,655	99.76%	500	254(scaffolds)	100%
11_6	11,655	99.78%	500	231(scaffolds)	100%
11_8	11,655	99.77%	500	213(sacffolds)	100%
12_2	2,272	99.82%	500	500(scaffolds)	100%
12_3	2,272	99.78%	500	500(scaffolds)	100%
12_5	2,272	99.87%	500	500(scaffolds)	100%
12_6	2,272	99.80%	500	500(scaffolds)	100%

Table S3 The codon uses and the RSCU value in the *R. chingii* mitogenome

Amino acid	Codon	Frequency of use	RSCU	Amino acid	Codon	Frequency of use	RSCU
Phe	UUU	492	1.14	Tyr	UAU	252	1.26
	UUC	368	0.86		UAC	148	0.74
Leu	UUA	278	1.18	TER	UAA	66	0.81
	UUG	286	1.21		UAG	103	1.27
	CUU	289	1.22	His	CAU	224	1.28
	CUC	181	0.77		CAC	127	0.72
	CUA	244	1.03	Gln	CAA	241	1.32
	CUG	140	0.59		CAG	125	0.68
Ile	AUU	399	1.21	Asn	AAU	264	1.18
	AUC	314	0.96		AAC	183	0.82
	AUA	273	0.83	Lys	AAA	334	1.13
Met	AUG	318	1		AAG	259	0.87
Val	GUU	235	1.28	Asp	GAU	232	1.26
	GUC	147	0.80		GAC	136	0.74
	GUA	201	1.10	Glu	GAA	352	1.30
	GUG	149	0.81		GAG	188	0.70
Ser	UCU	278	1.40	Cys	UGU	126	1.04
	UCC	206	1.04		UGC	117	0.96
	UCA	224	1.13	TER	UGA	74	0.91
	UCG	170	0.85	Trp	UGG	225	1
Pro	CCU	228	1.37	Arg	CGU	151	1.05
	CCC	136	0.82		CGC	69	0.48
	CCA	188	1.13		CGA	166	1.16
	CCG	115	0.69		CGG	123	0.86
Thr	ACU	183	1.22	Ser	AGU	177	0.89
	ACC	144	0.96		AGC	139	0.70
	ACA	163	1.09	Arg	AGA	227	1.58
	ACG	108	0.72		AGG	124	0.87
Ala	GCU	225	1.43	Gly	GGU	242	1.23
	GCC	142	0.90		GGC	105	0.53
	GCA	152	0.96		GGA	274	1.39
	GCG	112	0.71		GGG	166	0.84

Table S4 Specific characteristics of MTPTs in the *R. chingii* mitogenome

MTPT	Aligened length (bp)	Mitogenome		Plastome		Contained genes
		Start	End	Start	End	
1	465	10,159	9,694	84,906	85,371	patial-rpl2[2]
2	162	36,394	36,232	30,344	30,510	trnD-GUG[2]
3	180	36,876	37,056	45,239	45,419	trnS-GGA[1, 2]
4	187	37,062	37,249	45,994	46,186	partial-rps4[2]
5	360	59,198	58,838	102,321	102,681	partial-ycf68[2], partial-trnL-CAA[2]
6	290	65,659	65,369	476	766	-
7	864	101,618	100,754	19,726	20,590	partial-rpoC1[2]
8	359	144,931	145,290	106,799	107,154	-
9	137	208,390	208,253	1,370	1,507	-
10	124	209,749	209,873	92,997	93,121	-
11	2829	214,094	211,265	102,977	105,806	trnI-GAU[2], trnA-UGC[2]
12	232	219,103	219,335	21,268	21,500	partial-rpoC2[2]
13	262	292,436	292,174	101,616	101,879	-
14	4512	309,629	305,117	94,180	98,692	trnL-CAA[1,2], rps7[1,2], ndhB[1,2]
15	132	345,919	345,787	4,269	4,401	-
16	288	399,455	399,743	101,060	101,348	-
17	1167	430,152	428,985	85,381	86,548	partial-ccmC[1], rpl23[1,2], trnI-CAU[2]
18	2354	464,710	462,356	23,311	25,665	partial-rpoB[2]
19	1206	465,904	464,698	21,619	22,825	-
20	653	632,530	631,877	66,587	67,251	petG[2]
21	111	632,619	632,508	66,164	66,275	-
22	1401	634228	632,827	64,773	66,173	psbJ[1,2], psbL[1,2], psbF[1,2], psbE[1,2]
23	242	700,268	700,026	1,162	1,405	partial-psbA[2]
24	172	780,138	779,966	38,695	38,866	partial-psaB[2]

Note: [1], gene in mitogenome; [2], gene in plastome; partial-, gene fragments exist in MTPTs.

Table S5 Shared genes and endemic genes in the three species (*R. chingii, P. chinensis, A. indica*)

<table>
<tr><th></th><th colspan="2">mitogenome</th><th colspan="2">plastome</th></tr>
<tr><th></th><th>share gene</th><th>Endemic gene</th><th>share gene</th><th>Endemic gene</th></tr>
<tr><td>R. chingii</td><td rowspan="3">atp1, atp4, atp6, atp8, atp9, ccmB, ccmC, ccmFc, ccmFn, cob, cox1, cox2, cox3, mttB, matR, nad1, nad2, nad3, nad4, nad4L, nad5, nad6, nad7, nad9, rpl2, rpl5, rpl10, rpl16, rps3, rps4, rps12, rps13, rps14</td><td>rps10, rps7, rpl23, sdh3, sdh4, ndhB, psbE, psbF, psbJ, psbL</td><td rowspan="3">clpP, rpl2, rpl14, rpl16, rpl20, rpl22, rpl23, rpl33, rpl36, rps2, rps3, rps4, rps7, rps8, rps11, rps12, rps14, rps16, rps18, rps19, ycf2</td><td>accD, atpA, atpB, atpE, atpF, atpH, atpI, ccsA, cemA, matK, ndhA, ndhB, ndhC, ndhD, ndhE, ndhF, ndhG, ndhH, ndhI, ndhJ, ndhK, petA, petB, petD, petG, petL, petN, psaA, psaB, psaC, psaI, psaJ, psbA, psbB, psbC, psbD, psbE, psbF, psbH, psbI, psbJ, psbK, psbL, psbM, psbN, psbT, psbZ, rbcL, rpl32, rpoA, rpoB, rpoC1, rpoC2, rps15, ycf1, ycf3, ycf4, ycf15, ycf68</td></tr>
<tr><td>P. chinensis</td><td>rps10, sdh3, sdh4</td><td>atpA, atpB, atpE, atpF, atpH, atpI, cemA, infA, petA, petB, petD, petG, petL, petN, psaA, psaB, psaI, psaJ, psbA, psbB, psbC, psbD, psbE, psbF, psbH, psbI, psbJ, psbK, psbL, psbM, psbN, psbT, psbZ, rbcL, rpoA, rpoB, rpoC1, rpoC2, ycf3, ycf4</td></tr>
<tr><td>A. indica</td><td>-</td><td>accD, matK, rps15, ycf1</td></tr>
</table>

Table S6 Characterization of the sequence matrices used for organellar phylogenetic inference.

	Total number of sites	Sites with alignment gaps or missing data	Invariable sites	Singleton variable sites	Parsimony informative sites
Mitogenome	29,713	9.03%	26,690	2,363	660
Plastome	82,350	26.72%	67,326	10,599	4,465

Table S7 Characterizations of mitogenomes of 11 Lamiales species.

Species	Family	Total length (bp)	Gene number	CDS number	GC%
Pedicularis chinensis	Orobanchaceae	225,612	62	37	44.4
Pedicularis kansuensis	Orobanchaceae	273,598	63	37	44.3
Castilleja paramensis	Orobanchaceae	495,499	68	36	43.5
Aeginetia indica	Orobanchaceae	420,362	55	33	43.5
Rehmannia chingii	Orobanchaceae	783,161	77	47	44.8
Rehmannia glutinosa	Orobanchaceae	547,032	71	44	45.0
Salvia miltiorrhiza	Lamiaceae	499,236	69	42	44.4
Utricularia reniformis	Lentibulariaceae	857,234	68	40	44.0
Aragoa cleefii	Plantaginaceae	365,824	55	34	44.9
Dorcoceras hygrometricum	Gesneriaceae	510,519	60	29	43.3
Osmanthus fragrans	Oleaceae	563,202	74	44	44.6

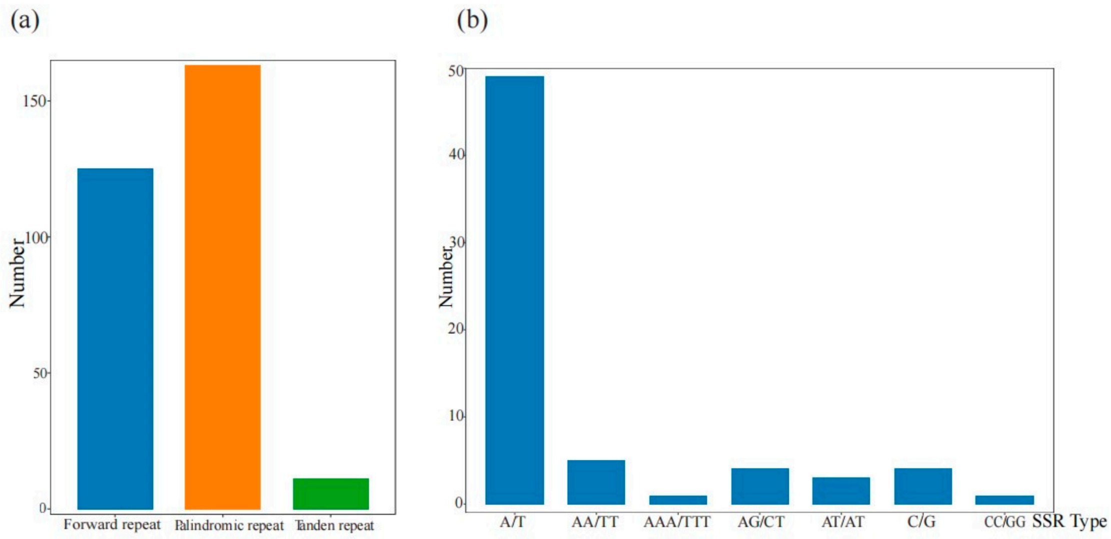

Figure S1. Repeat analysis of *R. chingii* mitogenome. (a) Repeat sequence statistics in the *R. chingii* mitogenome. (b) SSR statistics in the *R. chingii* mitogenome.
